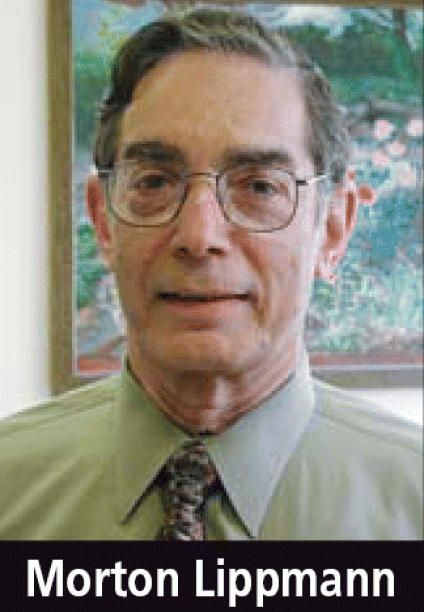# Guest Editorial: Norton Nelson’s Legacy: The Science of Environmental Health

**DOI:** 10.1289/ehp.114-1367851

**Published:** 2006-02

**Authors:** Seymour Garte, Bernard D. Goldstein, Paul Lioy, Morton Lippmann

**Affiliations:** University of Pittsburgh Cancer Institute, Pittsburgh, Pennsylvania; University of Pittsburgh, Pittsburgh, Pennsylvania; Environmental and Occupational Health Sciences Institute, Piscataway, New Jersey; New York University School of Medicine, Tuxedo, New York, E-mail: lippmann@env.med.nyu.edu

The field of environmental health science is now a recognized and respected branch of science in American and worldwide academia. One of the pioneers who had a major role in the establishment of environmental health as a true scientific discipline was Norton Nelson, the founder and first chair of the Department and the Institute of Environmental Medicine at New York University (NYU), which now bears his name. Among his many accomplishments, he played a leading role in the formulation of legislation that created the National Institute of Environmental Health Sciences (NIEHS) (Lippmann 2001). We would like to remind readers of some of the basic principles that Nelson and other pioneers of the new discipline invoked in order to establish environmental health science as an important discipline and to overcome barriers to acceptance of the field as a true science by those in other disciplines.

By early in the second half of the twentieth century, it was apparent that the issues raised by the effects of the chemical and physical environment on human health could not be addressed by a single branch of scientific research. Instead, such issues transcend the variety of quite disparate and nonoverlapping fields, each with separate and diverse methodologies, conceptual frameworks, and scientific cultures. These included toxicology, physiology, pharmacology, industrial hygiene, occupational medicine, epidemiology, exposure assessment, radiation and health physics, analytical chemistry, organic chemistry, biochemistry, ecology, and others that developed more recently, such as molecular biology and exposure science. The multidisciplinary nature of the issues and methodologies involved in environmental health science leads to the question of how the field and the scientists working in it can be defined. When Nelson began to organize his department at NYU, there was no clear answer to this question.

Especially after the peaking of interest in ecology and the environment that followed the first Earth Day in 1970, some colleges and universities, established “environmental studies” as an academic theme. The approach in many such programs was to combine ecology with social and political aspects of air and water pollution, as well as a sprinkling of toxicology, industrial hygiene, and statistics.

Nelson’s approach was different. While acknowledging that environmental health science by its nature must be an interdisciplinary field, he insisted that the practitioners of research and teaching in his department first, be highly trained in a basic science (e.g., biology, engineering), and then, as a second layer of training and expertise, acquire some significant degree of knowledge of other areas included in the realm of environmental health sciences. Nelson’s mission was to assemble—first within his own institute and then throughout the country—a cadre of specialists in epidemiology, toxicology, engineering, chemistry, and so on who would interact with each other and devote their expertise to solving environmental health problems.

As far as training new scientists for careers in environmental health science is concerned, Nelson always maintained that such training programs should be based on in-depth training in one of the major environmental health science specialties with electives in the others. For Nelson, an environmental health science researcher could be, for example, a toxicologist who is familiar with the techniques and current issues in environmental epidemiology and some health physics, and could understand a seminar on exposure assessment. Nelson’s own expertise in so many fields left many who knew him wondering what his original scientific training had been. In fact, his graduate degree was in biochemistry, a field in which he made significant contributions to the basic literature.

Nelson’s ideal was that every environmental health scientist should be on an equal footing with his/her peers in a chosen specialty, with the difference that he/she was also knowledgeable in several other fields also related to environmental health science. This vision has in fact come to pass, as can be seen in the many outstanding academic departments of environmental health science around the country and, of course, at the NIEHS itself.

Another important issue for environmental health science that Nelson tackled directly was that of social, political, and economic influences on the field. Although many branches of science interact with social forces, few do so as much as environmental health science. Nelson was quite clear and specific in his attitudes toward the role of social and other extrascientific forces in the conduct of environmental health science research. Because of the high economic and social impact that the results of environmental health research could have, and the pressures that could be brought to bear on the scientists working in the area, Nelson was quite strict about the crucial role that objective, independent, and nonaligned scientific work must play in advancing our understanding of environmental health. He did not favor any social agenda, either environmentalist or antienvironmentalist, and always insisted that all conclusions and recommendations be based solely on objective data and results. Further, decisions about what research should be done, how the results should be analyzed and interpreted, and how conclusions should be drawn must depend solely on basic mechanistic and scientific considerations and principles and not be subject to sponsors’ approval before publication in the peer-reviewed literature.

Nelson insisted on objectivity and independence from social agendas because he knew that only a reputation for honesty, integrity, and utmost objectivity would allow widespread acceptance of the results and conclusions of the research being conducted. Certain investigators and even institutions working in the field became known as pro-union or pro-environmentalist or pro-industry, and their credibility did not approach that of Nelson’s and other similar academic departments.

We all were trained and mentored by Norton Nelson prior to his death in 1990, and along with scientists throughout the world, we accept Nelson’s principles for research and training in environmental health science as the standard for the field. The origins of his well-accepted principles and ideas are often unknown to many people who use them; most people rarely even think about these principles that, not so long ago, needed to be articulated and defended. This is all to the good, for it shows the extent to which Nelson’s legacy has been accepted and absorbed into the standard operating principles of environmental health science as a scientific discipline.

## Figures and Tables

**Figure:**
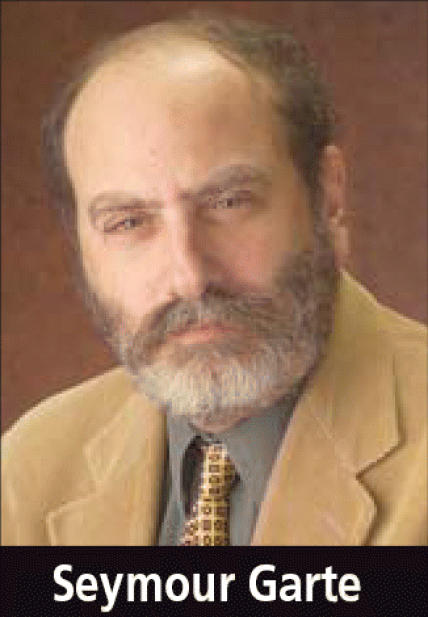


**Figure:**
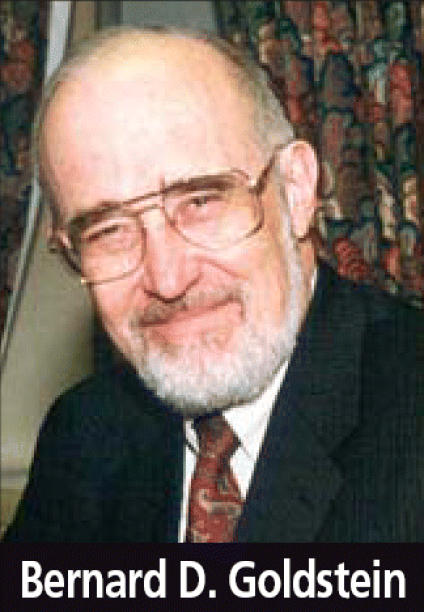


**Figure:**
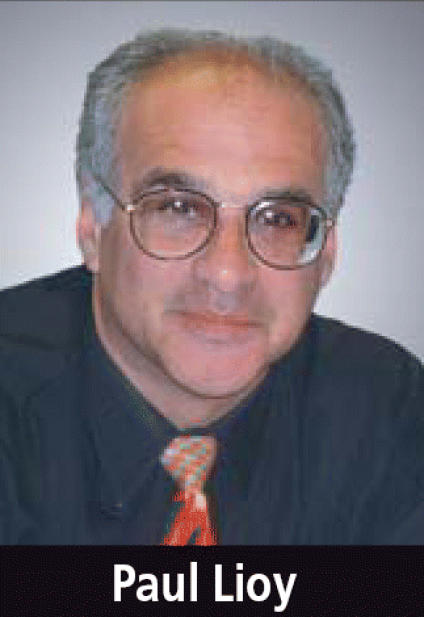


**Figure:**